# Genome-wide identification and evolutionary analysis of TGA transcription factors in soybean

**DOI:** 10.1038/s41598-019-47316-z

**Published:** 2019-08-01

**Authors:** Ihteram Ullah, Mahmoud Magdy, Lixiang Wang, Mengyu Liu, Xia Li

**Affiliations:** 1 Center for Agricultural Resources Research, Institute of Genetics and Developmental Biology, Chinese Academy of Sciences, Shijiazhuang, China; 2 University of Chinese Academy of Sciences, Beijing, China; 3 State Key Laboratory of Agriculture Microbiology, College of Plant Science and Technology, Huazhong Agricultural University, Wuhan, 430070 Hubei Province China; 4 Key laboratory of horticulture, plant biology, Huazhong Agricultural University, Wuhan, China; 5 Genetics Department, Faculty of Agriculture, Ain Shams University, Cairo, Egypt; 6 School of biological and chemical engineering, Panzhihua University, Panzhihua, China

**Keywords:** Phylogenetics, Phylogeny, Evolutionary genetics, Molecular evolution

## Abstract

The gain of function in genes and gene families is a continuous process and is a key factor in understanding gene and genome evolution in plants. TGACG-Binding (TGA) transcription factors (TFs) have long been known for their essential roles in plant defence in *Arabidopsis*, but their roles in legume symbiosis are yet to be explored. Here, we identified a total of 25 *TGA* (named *GmTGA1*-*GmTGA25*) genes in soybean. Through phylogenetic analysis, we discovered a clade of GmTGA proteins that appear to be legume-specific. Among them, two GmTGAs were unique by possessing the autophagy sequence in their proteins, while the third one was an orphan gene in soybean. *GmTGA*s were structurally different from *AtTGA*s, and their expression patterns also differed with the dominant expression of *AtTGA*s and *GmTGA*s in aerial and underground parts, respectively. Moreover, twenty-five *GmTGAs* showed a strong correlation among the gene expression in roots, nodules, and root hairs. The qRT-PCR analysis results revealed that among 15 tested *GmTGAs*, six were induced and four were suppressed by rhizobia inoculation, while 11 of these *GmTGAs* were induced by high nitrate. Our findings suggested the important roles of *GmTGAs* in symbiotic nodulation and in response to nitrogen availability in soybean.

## Introduction

To cope with the deficiency of nitrogen (N_2_) availability in soil, legumes develop specialized symbiotic organs, roots nodules, through association with nitrogen-fixing bacteria, called rhizobia. Root nodules make legumes capable of fixing atmospheric N_2_ as a nitrogen source for plants. This unique capability of fixing atmospheric N_2_ gives legumes a great advantage in growing in N_2_ deficient soils and hence are more successful in survival in the nutrient-stressed environment than other plants. However, the establishment of symbiosis between plant and rhizobia need a cascade of events in the roots of legumes^1^. This event is started with the release of flavonoids from the plant, which are sensed by rhizobia to trigger the production of node factor molecules^2^. These nod factor signals are perceived by the specialized receptors in root hair cells to activate a signal transduction cascade for rhizobial infection and nodule formation. Accordingly, a series of physiological, biochemical and morphological changes occur resulting in root hair deformation, the formation of infection thread that grows like a tunnel for rhizobial infection, simultaneous cell division mainly at the cortical cells of roots, and symbiotic nodule formation^3,4^. The primary function of the nodules is to create a mutually beneficial environment helping rhizobia to efficiently fix nitrogen.

Legumes that establish a symbiotic relationship with rhizobia, also need to deal with pathogens in natural soil rhizosphere^5^. These pathogens include but are not limited to oomycetes, fungi, and bacteria^6^. To cope with these pathogens, plants have maintained efficient transmembrane pattern recognition receptors (PRRs), which typically possess a leucine-rich repeat (LRR) or lysin motif (LysM) to recognize conserved microbial or pathogen-associated molecular patterns (MAMPs or PAMPs), such as flagellin (flg22)^4^. On the recognition of flg22, host plant induces P/MAMPs-triggered immunity (P/MTI) including Ca^2+^ influx to the cytoplasm, the production of reactive oxygen species (ROS) and the induction of defence-related genes^5^. Effector molecules of virulent pathogens usually suppress M/PTI and plant must activate effector-triggered immunity (ETI) through intracellular resistance proteins to against the invaders^7,8^.

Besides PTI and ETI, there is another mechanism which is known as systematic acquired resistance (SAR) whereby the *pathogenesis-related gene 1* (*PR1*) is induced by non-repressor of pathogenesis-related gene 1 (NPR1) and *TGA* TFs in *Arabidopsis*. The SAR defence response is triggered by elevated salicylic acid (SA), a phenolic plant growth regulator, through a SA-NPR1-TGA-PR1 signalling cascade at the sites of prime infection^6^. TGA transcription factors (TFs) are the members of bZIP family which is highly conserved in plants, animals, and micro-organisms. The *Arabidopsis* TGAs has 10 members and play crucial roles in disease resistance, stress mitigation, and flower development. These TGAs execute their activity by binding to *cis*-regulatory elements with TGACG as core recognition sequence and named as activation sequence-1 (as-1) motif^7^. Variations of this motif are present in promoters of various stress-related plant genes, such as *PR1* or *GLUTATHIONE-S-TRANSFERASE* (*GST*)^7^. Orthologs of *Arabidopsis* TGAs from other plant species also play roles in transcriptional regulation of the defence genes and protection against diseases, particularly in SAR. Because NPR1 cannot bind directly to the promoter region of *PR1*, TGAs are the core signalling components to interact with NPR1 and mediate activation of SAR. In *Arabidopsis*, TGA2, TGA3, TGA5, TGA6, and TGA7 constitutively interact with NPR1 in yeast and planta and TGA1 and TGA4 can bind to NPR1 in SA treated leaves^9^. Thus, all seven TGA TFs are involved in SAR and are the key players in the plant defence system.

The afore-mentioned mechanisms highlight that symbiosis and plant immunity go head-to-head in legumes during nodulation and the latter must get aside to let the symbiosis occur. Since some rhizobia do not elicit an obvious defence response, it has been proposed that rhizobia can suppress the host immune system to allow the infection and symbiosis establishment^8^. Alternatively, legume plants must continuously adapt to engage compatible interactions with symbionts and fight to control pathogens^10^. For some other rhizobial-plant interactions, a defence response may be elicited and is involved in determining host range or nodule formation^11^. It has been shown that rhizobial infection alters the expression of the genes involving both symbiosis and immune responses, including bZIP family genes in leguminous plants^12^. Some studies showed that plants keep some players that can play in both the teams simultaneously and can recognize both the friends and foes. For example, Nod Factor Perception (NFP) and LysM-Receptor-like Kinase 3 (MtLYK3) are involved in both symbiosis and disease resistance in *M*. *trunctula*^13^. Interestingly, two recent studies showed that rhizobial inoculation may interact with plant defence through SA-mediated systemic resistance. In *M*. *trunctula* and *Pisum sativum*, rhizobial nodulation greatly enhances plant resistance to powdery mildew through inducing systemic resistance and priming for powdery mildew-triggered SA accumulation^14^, while in soybean Gr.3 soil bacteria inhibit nodulation mainly by inducing *PR1* and *PR5* gene expression^15^. These results implicate the central role of SA and the SA signalling pathway in the crosstalk between nodulation and plant defence signalling pathways.

The role of SA in symbiotic nodulation has long been noticed. Exogenous application of SA exerts an inhibitory effect on indeterminate nodules but does not affect determinate nodule^16,17^. However, additional studies investigating the determinate nodule-forming soybean have reported an inhibitory effect of SA treatment on nodulation^18,19^. Reduction of endogenous SA levels by overexpression of *salicylate hydroxylase* (*NahG*) increased nodulation both in indeterminate (*M*. *truncatula*) and determinate (*L*. *japonicus*) legume species^20^, suggesting a complex role of SA in symbiotic nodulation of plants developing indeterminate and determinate nodules. Further genetic evidence demonstrated that nod factors (NFs) may suppress SA induced by bacteria^21^, revealing a potential mechanism by which rhizobial infection may suppress plant defence by modulating SA accumulation during nodulation. However, many key questions remain unaddressed, and these include how SA accumulation is regulated during nodulation, how nodulation signalling and SA signalling pathways interplay and so on. In this study, we report the identification and the systematic analysis of TGA TFs, candidate components of the SA signalling in soybean. Comparative analysis of these *TGAs* revealed structural diversity, expressional variations, and functional divergence. In contrast to *Brassicaceae* originated *AtTGAs*, the *GmTGAs* of *Fabaceae* origin have unique expression pattern with their predominant expression in underground parts (roots and nodules) compared to the aerial parts in *Arabidopsis*. The difference in the expression patterns of TGA TFs in *Arabidopsis* and soybean implicate that TGA TFs might have gained an extra function as nodule regulators in soybean. Thus, our findings suggest the unique role of TGA TFs in soybean nodulation which can be further explored in legumes generally and in soybean specifically.

## Results

### Sequence analysis of soybean bZIP family

**Figure 1:**
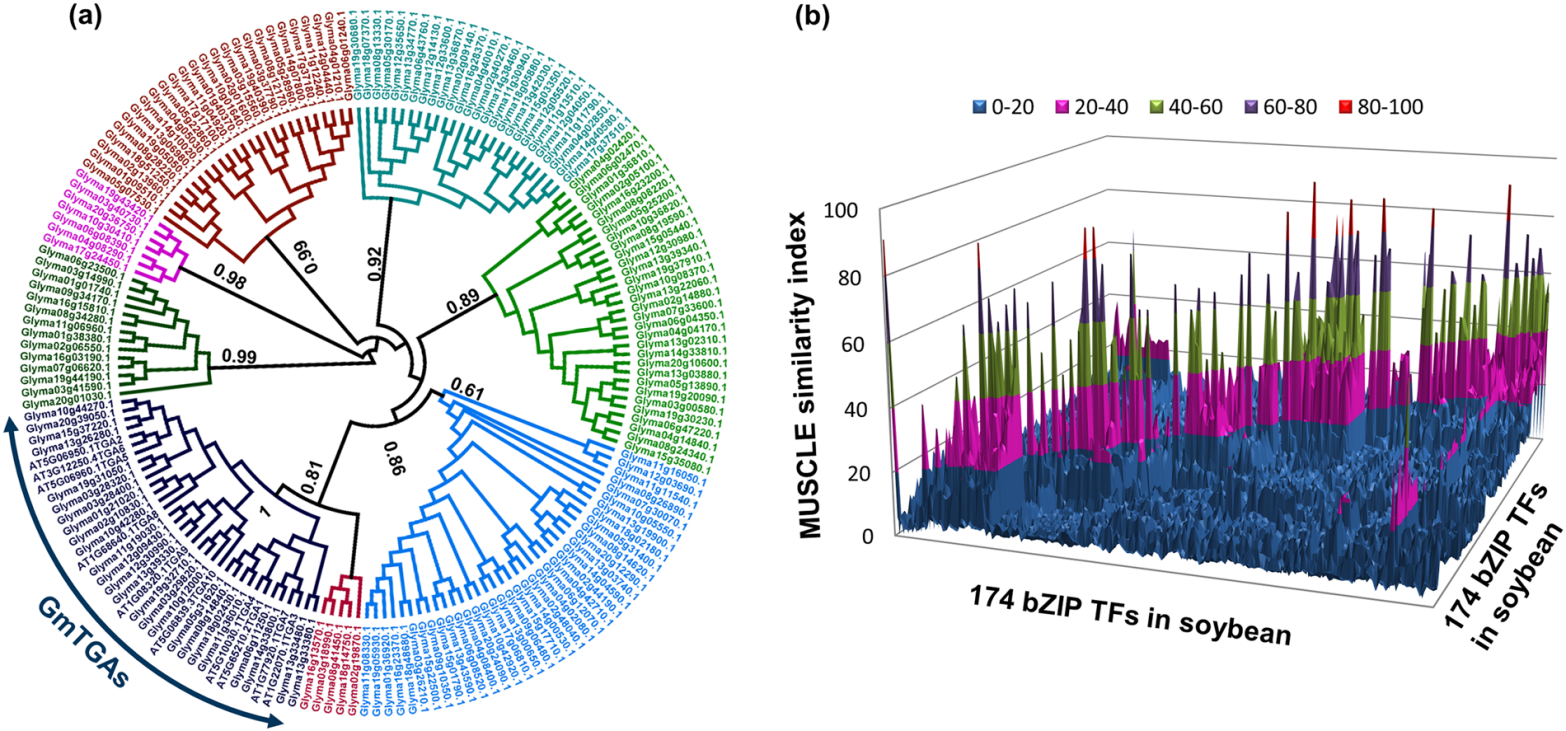
Phylogenetic analysis of soybean bZIP family of transcription factors. (**a**) Soybean bZIP TFs were divided into eight different groups based on FastTree. The last group which is colored as dark blue is comprised of the GmTGAs. *Arabidopsis* TGAs were used to extract the total TGAs from the soybean genome. (**b**) Similarity among bZIP TFs of the soybean genome using MUSCLE alignment values. The diagonals with 100 MUSCLE values (the same proteins that are compared) were not considered for plotting in the graph. Most of bZIP TFs had a similarity value less than 20 which show the diversity of bZIP in soybean.

Since *TGA* transcription factors belong to the bZIP protein family, we first accessed the browse gene family function of the Soykb database and selected the bZIP TF family to find the members of the bZIP family in the soybean genome. One hundred seventy-four bZIP TFs with a total of 366 variants were found, but for the sake of simplicity, one variant per gene was used for further analysis. Based on phylogenetic analysis of amino acid (AA) sequences, we found that bZIP TFs were very diverse in structure and formed eight groups in total (Fig. 1a). The structural diversity of these bZIPs suggests the diverse functions of these genes in soybean. The structural diversity of bZIP in soybean is also evident from the similarity matrices of MUSCLE analysis whereby the similarity among most of the GmbZIPs is less than 20 (Fig. 1b). GmbZIPs are distributed across 20 linkage groups with a median of 11 genes per chromosome (Supplementary Fig. 1). Chromosomes 02, 03, 04, 06, 08, 11 and 12 retained 11 genes of the bZIP family, while chromosome 13 have retained 16 genes followed by 12 genes by chromosome 19, and chromosome 20 had retained the least number (3) of bZIPs. The structural diversity and distinct alignment of these TFs suggest that there may be numerous sub-groups in GmbZIP with different functions or some of the members even might have gained different features from their ancestors.

### Protein properties and sequence analyses of GmTGAs

Since we were interested in TGA TFs (a subgroup in bZIP family), we downloaded the AA sequences of the known ten TGA TFs in *Arabidopsis* and then aligned them with the 174 soybean bZIP TFs to find out all the TGA TFs in soybean. Twenty-five proteins out of 174 bZIP TFs were aligned with AtTGAs (Supplementary Fig. S2) and were divided into the same clade with *Arabidopsis* TGA TFs (Fig. 1a). Thus, we identified a total of 25 TGA TFs in the soybean genome, which were named from *GmTGA1* to *GmTGA25* according to their position on chromosomes in the soybean genome and were used for further analyses.

**Figure 2:**
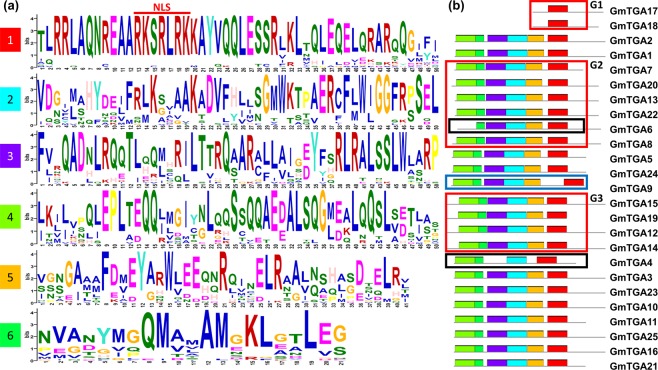
Conserved motifs in GmTGAs using MEME-suite. (**a**) The conserved motifs among GmTGA proteins. The first motif covers the bZIP domain, and the nuclear localization signals (NLS) is shown by drawing a red line on the top of the region. The rest of the five sequences lies in the second domain indicates high diversity of the functional domain among the GmTGAs. (**b**) The distribution of motifs along with the protein sequences. Group 1 (G1) highlights the first two GmTGAs as pseudo-genes that have retained the first motif (bZIP domain) only but have lost the other domains during evolution. Twenty-one GmTGAs share all the six motifs, while GmTGA4 and GmTGA6 (surrounded by black frame) have lost two and one motifs, respectively. GmTGA9 (surrounded by a blue frame) has gained or duplicated some region between motif 1 and motif 5 which is a deviation from the other GmTGAs. G2 and G3 encircle the groups which have lost the region between motif 3 and 6 as compared to the rest of GmTGAs.

All the 25 GmTGAs were then subjected to MEME online tool (http://meme-suite.org/) to find the common motifs among these proteins. The analysis was performed using “searching for six motifs” while the rest of the setting were kept as default. Majority of these GmTGAs contained 6 motifs except GmTGA17 and GmTGA18 of Group 1 (G1), GmTGA6 in G2 and GmTGA4 in G3 (Fig. 2b). As shown in Fig. 2a, motif-1 was highly conserved in these GmTGA proteins, which is responsible for DNA binding and contains nuclear localization signals (NLS). This motif is also rich in glutamine (Q), which is usually associated with dimerization of the proteins. Motif-3 and Motif-4 were also rich in Q with motif-4 as the Q-richest motif. The presence of Q rich motifs in GmTGAs suggested that these proteins could interact with a variety of other proteins, thereby making GmTGAs better candidates to integrate various biological processes in soybean. Motif-2, motif-5 and motif-6 were highly variable among the GmTGA proteins which might be responsible for different biological functions in these GmTGAs. The presence of highly variable motifs suggested a wide range of functional diversity for GmTGAs. Also, obvious contractions were observed in the proteins structures between motif 3 and 6 in groups G2 and G3 which are probably in the result of segment deletions in these genes. G1 represents the possible group of pseudogenes, while GmTGA6 and GmTGA4 proteins had lost one and two motifs, respectively (Fig. 2b). The loss and gain of a portion of AA sequences may lead to the expansion and contraction of the proteins resulting in changes of the protein functions, especially if the event occurs within the domain. Furthermore, the sequences were subjected to hfAIM (http://bioinformatics.psb.ugent.be/beg/software) to search autophagy “autophagy-associated atg8-interacting motifs”^22^ and found that two of the GmTGAs (GmTGA3 and GmTGA23) contains the autophagy sequence (MTQFDDI) in their protein sequences.

### Evolutionary and phylogenetic relationship between *Arabidopsis* and soybean TGAs

**Figure 3:**
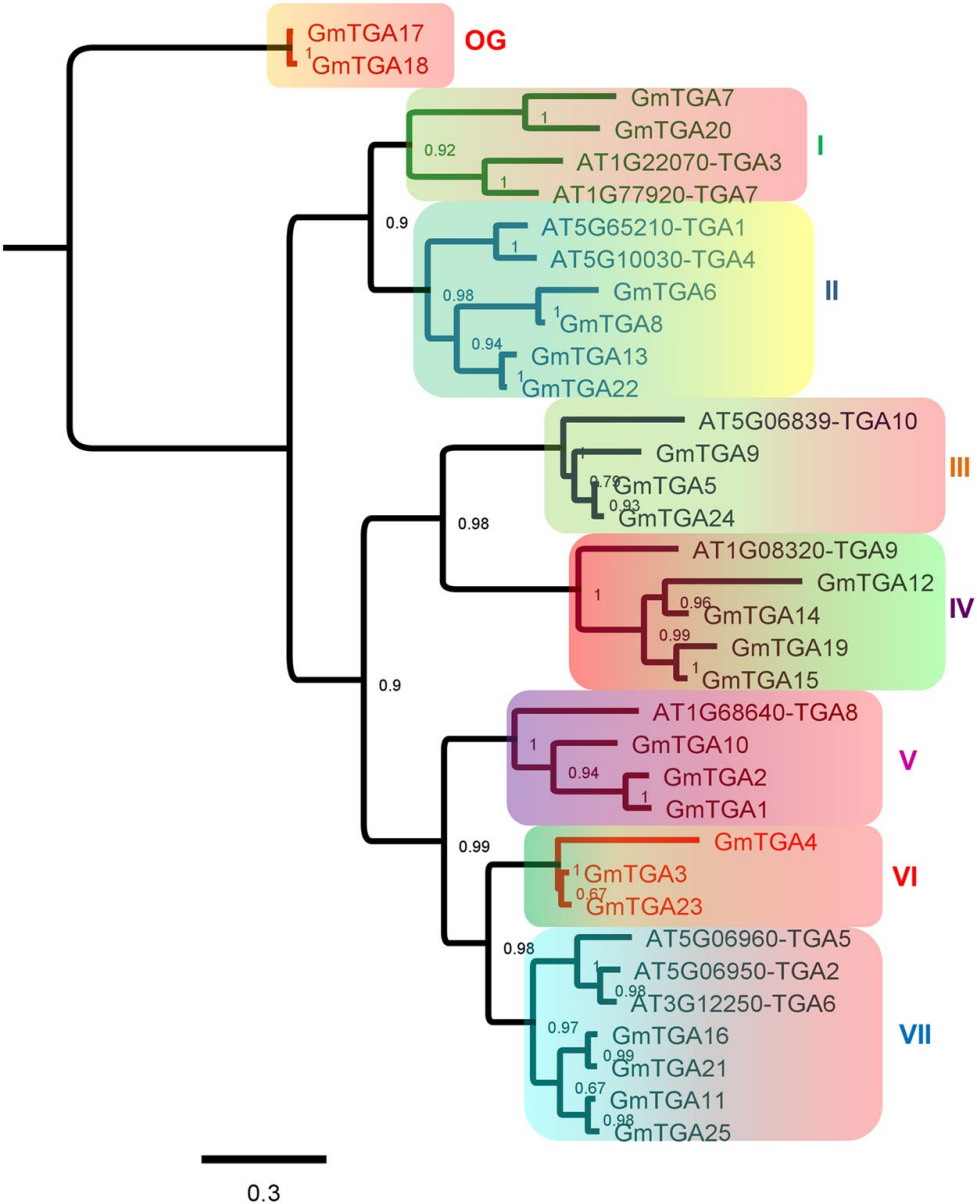
Phylogenetic relationship between GmTGAs and AtTGAs. All the 10 AtTGAs were grouped with the members of 25 GmTGAs in different clades, except Clade-VI which had no AtTGAs and the group was specific to soybean. The Pseudo-genes were grouped as an out-group in the tree. Node values are the confidence levels for duplication events between genes or gene clades, while the scale bar shows the substitutions per site.

To analyze the phylogenetic relationship of these GmTGAs with AtTGAs, we aligned the protein sequences of all the 25 GmTGAs with 10 AtTGAs using MAFFT, and a phylogenetic tree was constructed using FastTree plugin in Geneious. The alignment and subsequent tree construction were inevitable to characterize the GmTGAs based on AtTAGs roughly. As shown in Fig. 3, two pseudo-genes were grouped into a separate clade and were considered as out-group. The tree was rooted based on the out-group to hierarchically group the genes based on evolution relationship from older to newer. AtTGA3 and AtTGA7 were grouped with 2 GmTGAs in Clade-1, AtTGA1 and AtTGA4 were grouped with 4 GmTGAs in clade-II, AtTGA9 and AtTGA10 were grouped with 3 and 4 GmTGAs in clade-III and IV, respectively. It is noteworthy that AtTGA9 and AtTGA10 evolve through parallel evolution in *Arabidopsis* as compared to the other counterparts which were evolved through gene duplication, and GmTGAs in the respective groups followed the same pattern. AtTGA8 was grouped with 3 GmTGAs in clade-V, while AtTGA2, AtTGA5 and AtTGA6 were grouped with 4 GmTGAs in clade-VII. Clade-VI was comprised of 3 GmTGAs (GmTGA3, GmTGA4 and GmTGA23), which were not grouped with any AtTGAs, and two of them contains the autophagy sequence in their proteins, making them unique. We also retrieved the gene duplication data for 17 GmTGAs from PLAZA and found that all of them had gone through gene duplication event. Some of them (i.e. GmTGAs of clade-I and II) may have duplicated for three times because six copies were found for these genes (Supplementary Table. S1).

For in-depth analysis, the *Arabidopsis* and soybean TGA sequences were MAFFT aligned and were presented in Supplementary Fig. S3. The GmTGA17 and GmTGA18 were out-grouped and only showed similarity to the rest of the sequences by its bZIP domain, while the second domain (which is shown as DOG1 domain by Pfam and Prosite) was lost during evolution (Supplementary Fig. S3). Soybean TGAs were far different from *Arabidopsis* TGAs in protein structures and the reason probably is the evolutionary distance between the two species. Clade-III, IV, and VII are the obvious examples of the protein structural diversity between soybean and *Arabidopsis* TGAs (Supplementary Fig. S3).

### A sub-clade of GmTGAs is legume-specific

**Figure 4:**
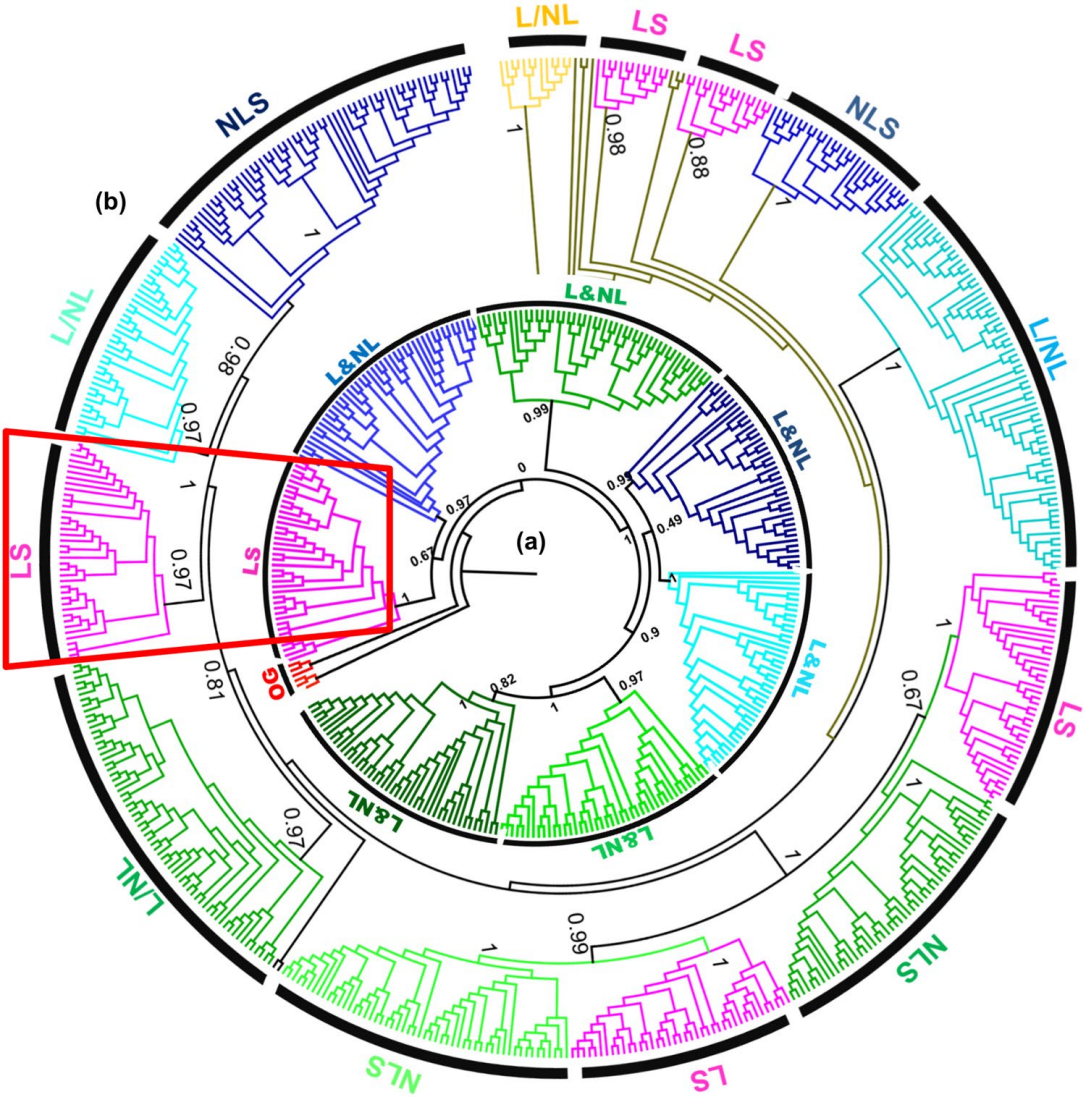
Phylogenetic trees of legumes and non-legumes TGA related TFs. (**a**) Phylogenetic tree of 303 sequences retrieved from NCBI has been divided into seven clades with a legume-specific clade (highlighted in purple colour). (**b**) The phylogenetic analysis of 539 TGA related TFs from different crops has yielded a total of 13 clades. Five of the clades are legume-specific (LS), four of them are non-legume specific (NLS), while the rest of the four have both the legumes and non-legumes (L/NL). The group that has been highlighted is the one that repeated itself in both trees with a constant number of genera and *spp* and is evolved in legumes specifically.

To further understand the evolutionary and phylogenetic relationship of GmTGAs, we blasted the protein sequences of all the 25 GmTGAs, and top 30 sequences for each query were selected and were used for evolutionary analysis among TGAs. A total of 750 sequences were retrieved from NCBI (https://www.ncbi.nlm.nih.gov) and the total number of these TGA homologs were then reduced to 268 by deleting the duplicates and poorly aligned sequences. We then aligned all the protein sequences including the 25 GmTGAs and 10 AtTGAs using MAFFT plugin in Geneious 11, and a phylogenetic tree was constructed using FastTree. The tree was grouped into seven clusters along with an out-group. As shown in Fig. 4a, GmTGAs were distributed across all the clades, while AtTGAs were distributed in six groups. Interestingly, all of TGAs in group LS (purple coloured and encircled in Fig. 4a) are legume TGAs, suggesting that this group of TGAs might be legume-specific. All the three unique GmTGAs (clade-VI, Fig. 3) were present in the legume-specific group which was comprised of 39 members from 16 species and 12 genera. The fact that these unique GmTGAs formed a separate clade with other legumes TGAs which is differentiated from TGAs in other plant species (Fig. 4a,b) suggests that these group of TGAs are highly likely legumes specific. Indeed, 32 out of 39 members of this TGA group contain the autophagy sequences (http://bioinformatics.psb.ugent.be/beg/software), supporting the notion that these TGAs are specifically evolved in legumes.

To confirm whether this group is specific to legumes, we blasted the 10 AtTGAs along with 25 GmTGAs and retrieved a total of 1050 sequences from legumes and non-legumes and finally had a set of 539 sequences including 35 reference sequences (10 AtTGAs and 25 GmTGAs) after deleting the duplicates and poorly aligned sequences. A tree was constructed and generated using MAFFT as an alignment and FastTree as phylogeny tool with the sequences clustered into 13 clades (Fig. 4b). Further close observation revealed that most of the clades contained either legumes or non-legumes. The legume-specific group that was identified in Fig. 4a, made a separate clade in Fig. 4b without losing or gaining any members, species or genera. The common ancestor for LS group in both trees (the encircled clade) was *Lupinus angustifolius* suggesting that the group is evolved in Papilionoid legumes much later in the evolutionary history of Leguminosae.

### Expression patterns of *GmTGA* genes in soybean

**Figure 5:**
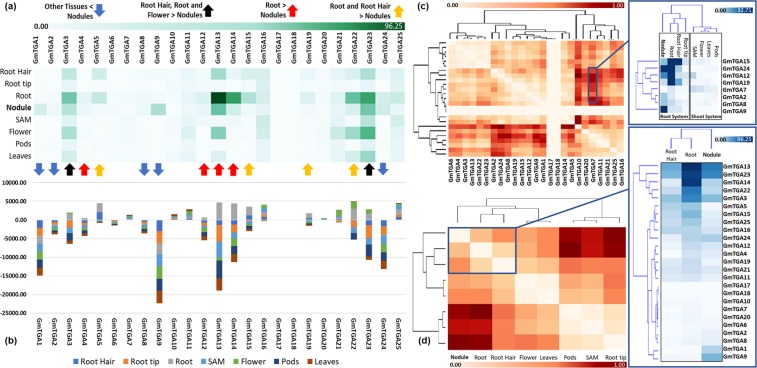
Tissue expression pattern of *GmTGAs*. (**a**) Majority of the *GmTGAs* genes were expressed highly in roots and nodules. (**b**) The relative expression of *GmTGA*s in different tissues to nodules. The positive and negative values showed that these *GmTGA*s genes were expressed at higher and lower levels in these tissues than in nodules, respectively. (**c**) Correlation of 25 *GmTGAs* based on their expression levels. Dark colour represents the strongly correlated, while the light colour represents poorly correlated *GmTGAs*. The highly correlated *GmTGAs* were extracted from the main figure based on their correlation, and their expression levels are shown in roots systems and shoot systems. They make two blocks by their high expression in root systems while their low expression in shoot systems. Dark colour represents high expression level, while the light colour represents the low expression level of the *GmTGAs* in the respective tissues. (**d**) Correlation analysis of the expression data of *GmTGAs* in seven tissues. There is a strong correlation among root, nodule, and root hair.

To explore the possible biological functions of these *GmTGA* genes, we retrieved expression data of *GmTGAs* from soybean eFP Browser and subjected to Orange statistical package and arranged for heatmap production and correlation analysis among *GmTGAs* based on their expression values. The heat map showed that majority of the *GmTGA* genes were expressed at higher levels in roots and nodules than other plant parts (Fig. 5a), and 3 of them were also highly expressed in other parts of plants (i.e., *GmTGA10* in leaf and *GmTGA11*, *GmTGA21* in flower). We then analyzed the relative expression of *GmTGAs* in different tissues compared with that in nodules (Fig. 5b). In comparison to *Arabidopsis*, only 3 of 10 *AtTGAs* were expressed highly in roots while the rest of 7 were expressed higher in aerial parts of the plants (Supplementary Fig. S4). The organ-specific changes in the expression patterns of *GmTGAs* indicate that *GmTGAs* might have gained new function(s) in underground parts in soybean during evolution.

Since nodule symbiosis is a prominent trait in soybean and there were some commonalities in the expression patterns of the genes between roots and nodules, we hypothesized that some of these *GmTGA* genes might be involved in the process of symbiotic nodulation. Interestingly, it was found that only a small portion of the total genes expressed in roots and nodules are either specific to roots or nodules, while the rest of the genes were co-expressed in both roots and nodules (Supplementary Fig. S5a). To investigate whether there is any correlation between the expression of the genes and two organs, we correlated the expression data of 25 *GmTGAs* and found that the expression of *GmTGAs* in soybean roots was highly correlated with nodules as well as with root hair (Supplementary Fig. S5b). Further analysis showed that these *GmTGA* genes were separated in different blocks based on their expression correlation in roots and nodules (Fig. 5c,d). Also, the expression of some of the genes are higher in underground plant tissues as compared to the aerial plant tissues in soybean (Fig. 5c). Notably, there was no expression data found for *GmTGA17* and *GmTGA18* in Plant Transcription Factor Database (http://planttfdb.cbi.pku.edu.cn/index.php), and there were no genes at these chromosomal locations in the phytozome database. It is likely that these two genes are pseudogenes and were lost during genome evolution. Based on these expression patterns and correlation analysis results, fifteen of these *GmTGAs* were selected for further expression validation.

### *GmTGAs* are responsive to rhizobia inoculation

**Figure 6:**
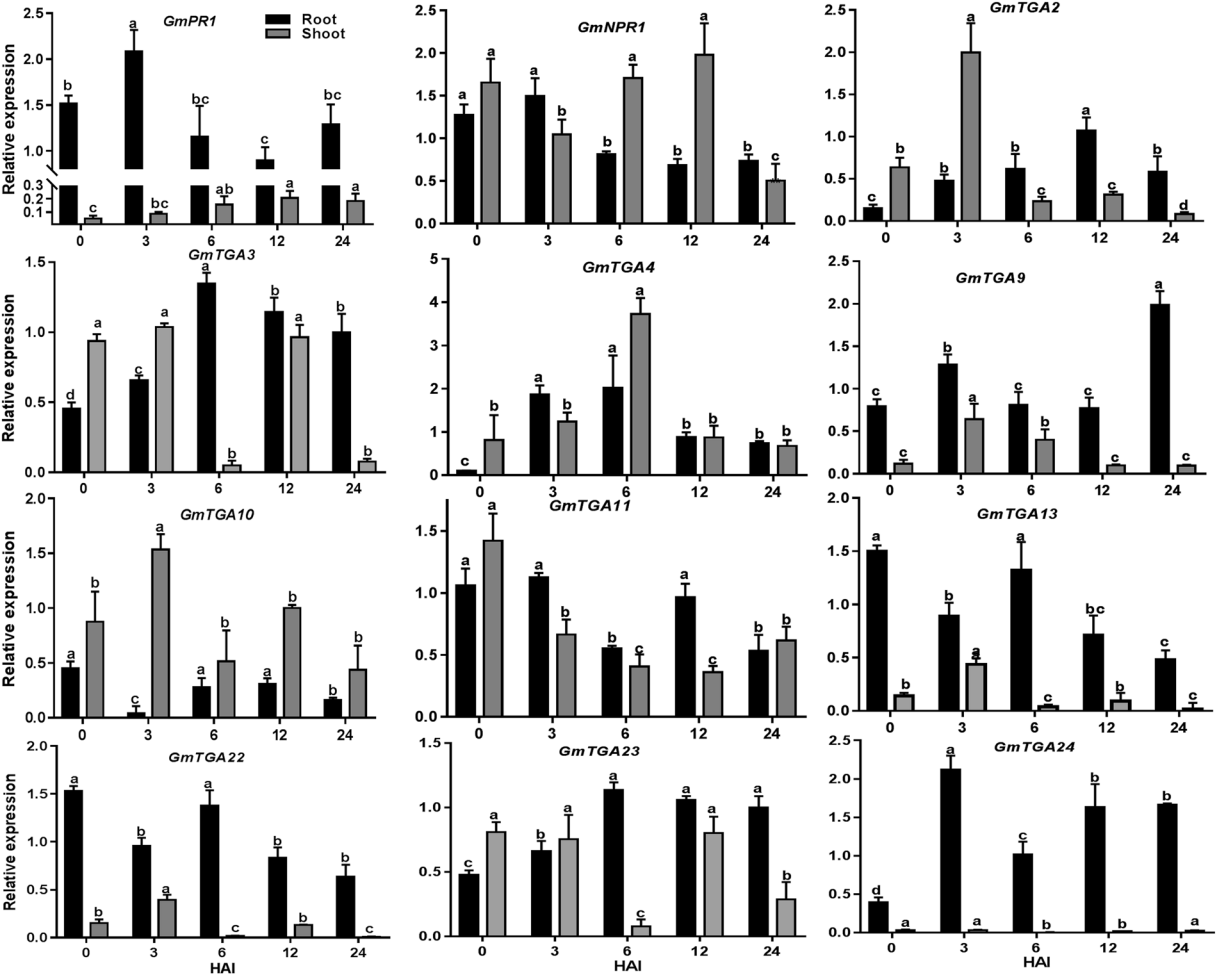
The expression of *GmTGAs*, *GmPR-1* and *GmNPR1* to rhizobia inoculation. The roots and shoots materials were collected at the specified time points and the abundance of gene transcripts was quantified by qRT-PCR. The roots were inoculated with *Bradyrhizobium diazoefficiens* USDA110 with 0.08 OD_600_. Error bars represent standard error of the mean (SEM), black bars represent the expression of *GmTGAs* in roots, while grey bars represent the expression levels in shoots. One way ANOVA was applied independently to the expression pattern of genes in roots and shoots. Bars sharing the same letters are non-significant while bars with different letters are significantly different in expression. HAI = hours after inoculation.

To validate the expression patterns of the selected *GmTGAs* in response to rhizobia, we collected the samples at the specified time points and performed qRT-PCR using *GmPR1* and *GmNPR1* as positive controls. In the root, *GmPR1* was induced at earlier time points of rhizobia infection and restored to the approximately basal levels during prolonged inoculation of rhizobia, while *GmNPR1* expression was suppressed by rhizobia inoculation. In comparison, these *GmTGAs* were differentially expressed in response to rhizobia inoculation. Among them, *GmTGA2*, *GmTGA3*, *GmTGA4*, *GmTGA9*, *GmTGA23* and *GmTGA24* were induced in the root, while *GmTGA10*, *GmTGA11*, *GmTGA13* and *GmTGA22* were suppressed by rhizobia within the 24 hours (Fig. 6). All the three members (*GmTGA3*, *GmTGA4* and *GmTGA23*) from the legume-specific clade were induced by rhizobia in the root, supporting the notion that they might have a role in nodulation.

It is noteworthy that the majority of the tested *GmTGA* genes were responsive to rhizobial infection in both root and shoot. Some of the *GmTGA* genes responded to rhizobia in both root and shoot, while several of them only displayed the response to rhizobia in the shoot. For example, *GmTGA4* was simultaneously induced by rhizobia in both root and shoot, while *GmTGA10* was downregulated in the root but upregulated in the shoot at 3 HAI (Fig. 6 and Supplementary Fig. S6a).

### Responsiveness of *GmTGAs* to nitrate treatment

**Figure 7:**
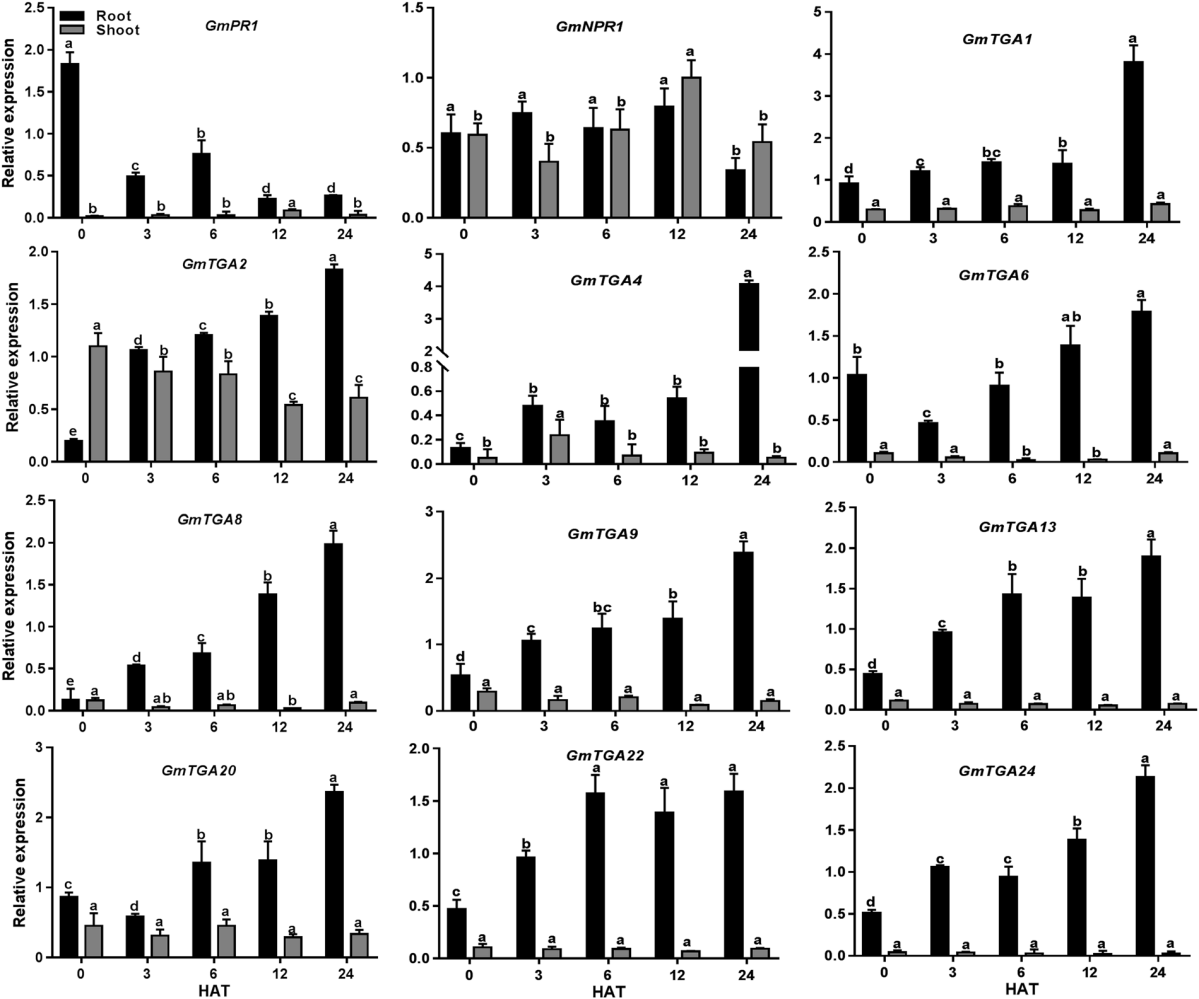
The expression of *GmPR1*, *GmNPR1* and *GmTGAs* in response to nitrate. The soybean plants were treated with nitrate for 24 hours and the samples were collected for gene analysis. The abundance of gene transcript was quantified by qRT-PCR at different time-points in roots and shoots. The roots were inoculated with *Bradyrhizobium diazoefficiens* USDA110 with 0.08 OD_600_. Error bars represent standard error of the mean (SEM), black bars represent the expression level in roots while grey bars represent the expression level in shoots. One way ANOVA was applied independently to the expression pattern of genes in roots and shoots. Bars sharing the same letters are non-significant while bars with different letters are significantly different in expression. HAT = hours after treatment.

We then analyzed the expression of the selected genes in response to nitrate treatment. As shown in Fig. 7, *GmPR1* expression was markedly suppressed by nitrate in the root, while the expression of *GmNPR1* remained unchanged in both root and shoot. In sharp contrast, all the tested *GmTGAs* except *GmTGA11*, *GmTGA23* and *GmTGA3* were gradually induced in response to high nitrate treatment and reached their highest expression levels within 24 hours. It is noteworthy that two members (*GmTGA*3 and *GmTGA23*) in the soybean-specific clade were suppressed by high nitrate, while *GmTGA4* was induced significantly. Majority of the *GmTGA* genes were expressed at higher levels in roots than in shoots, and the expression of these genes was less responsive to nitrate in the shoot.

### *GmTGAs* exhibit diverse organ responses to nitrate status

**Figure 8:**
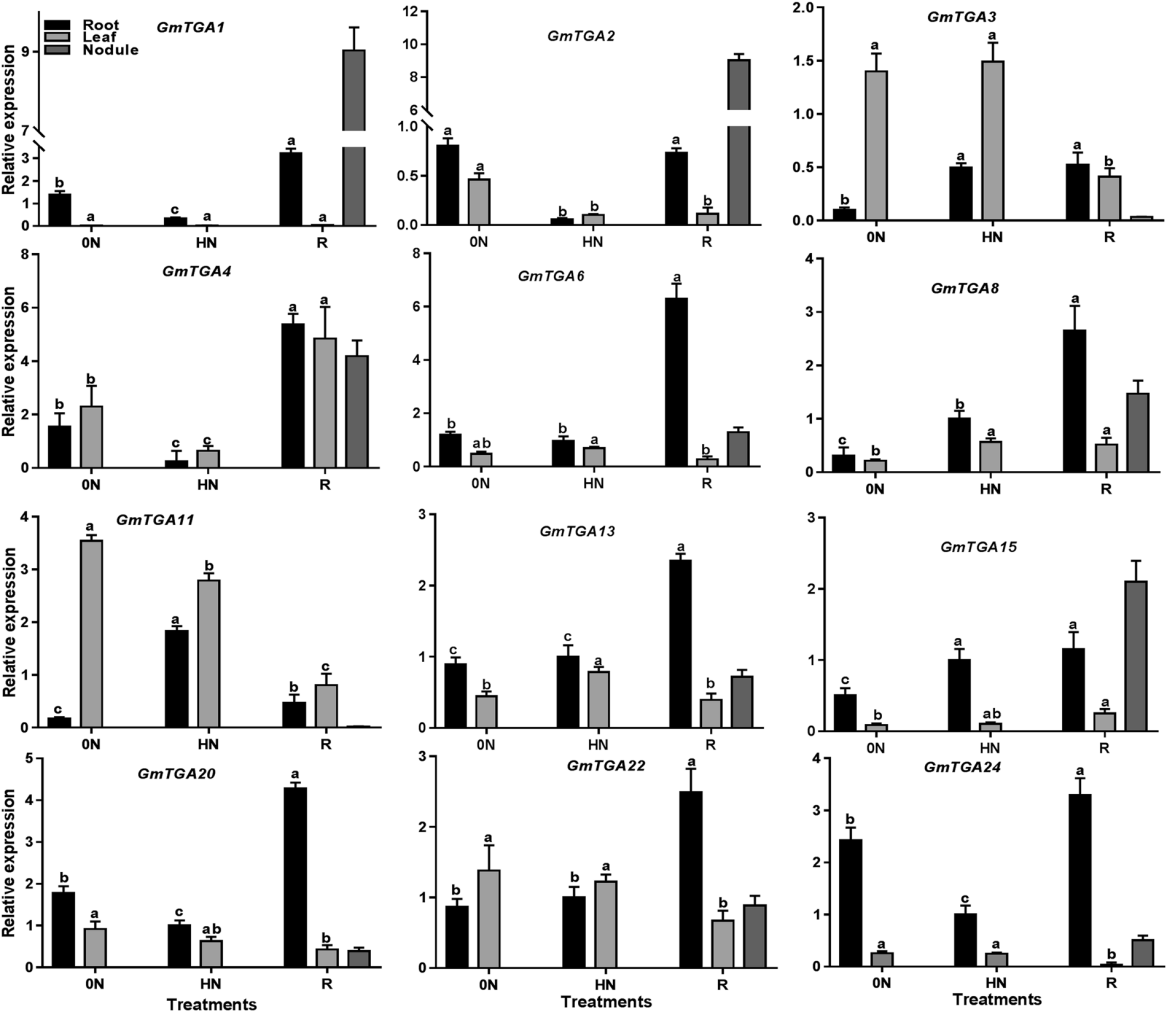
The expression of *GmTGAs* after rhizobia inoculation and high nitrate treatment. The materials were collected at 21 days after rhizobia inoculation (DAI) and high nitrate treatment. The abundance of gene transcript was quantified by qRT-PCR. Error bars represent standard error of the mean (SEM), black bars represent the expression level in roots, dark grey shows the expression level in nodules, while light grey colored bars represent the expression level in shoots. One way ANOVA was applied independently to the expression pattern of genes in roots and leaves. Bars sharing the same letters are non-significant while bars with different letters are significantly different in expression. The plants were grown under three different treatment condition, i.e. 0 N = plant grown under the nitrogen-free condition without rhizobia inoculation, HN = plants grown under high nitrate condition without rhizobia inoculation, R = plants grown under 0 N conditions but inoculated with rhizobia.

To investigate the possible roles of these *GmTGA* genes in different organs, we also analyzed the expression of these genes in shoot, root and/or nitrogen-fixing nodules at 21 days after nitrate treatments with or without rhizobial inoculation. As shown in Fig. 8, under no and nitrate without rhizobial infection, these *GmTGA* genes exhibited significant variations in the expression patterns in shoot and root. However, most of these genes showed the correlated expression in shoot and root under no or high nitrate although the expression levels of the genes varied. For example, *GmTGA24* expression level was higher in root than in shoot under both no and high nitrate treatments. In contrast, *GmTGA11* showed nearly the opposite expression pattern to *GmTGA24* at the organ level. Of the tested genes, expression of *GmTGA1* was only detected in roots under both no and high nitrate conditions. These results suggest diverse roles of the *GmTGA* genes in different organs in response to long term nitrate deficiency and high nitrate conditions.

In the plants inoculated with rhizobia under no nitrate condition, these *GmTGA* genes also exhibited varied expression patterns in the root, shoot and nodules (Fig. 8 and Supplementary Fig. S6c). Some *GmTGA* genes (*GmTGA6*, *GmTGA8*, *GmTGA9*, *GmTGA13*, *GmTGA20*, *GmTGA22* and *GmTGA24*) were expressed at much higher levels in root than in shoot and nodule, while several other *GmTGAs* (*GmTGA1*, *GmTGA2* and *GmTGA15*) exhibited higher expression in nodules. Overall, the majority of the *GmTGA* genes were expressed at the lower levels in shoot than in root and nodule. We also observed that several *GmTGA* genes were expressed at a very low level in shoot or nodule.

## Discussion

Legumes and rhizobia experience hostile interaction at the early infection stage and ultimately establish a mutualistic relationship to cope with the low nitrogen. Therefore, reprogramming of the plant defence system during rhizobial infection and symbiotic nodulation is the key driver for the legumes-rhizobia symbiosis. TGA TFs are the members of the bZIP family and play crucial roles in plant responses to microbial pathogens in plants, such as *Arabidopsis* and rice. It is recently reported that common bean (*Phaseolus vulgaris*) possesses a whole set of 78 bZIP (*PvbZIP*) genes, and the majority of these *PvbZIP* genes (59%) are highly expressed in root and nodule^23^. A previous study showed that 62% of the total *GmTGAs* studied were highly expressed in either roots or nodules^24^, while another transcriptomic study reported that 18 out 23 (74%) *GmTGAs* were highly expressed in roots and nodules as compared to the other plant tissues^25^. Several bZIP family genes, such as a light-regulated bZIP TF (*MtATB2*) in *M*. *trunctula*, *PsATB2* in pea and *ASTRAY* in *L*. *japonicus*, play important roles in symbiotic nodulation, nodule development and senescence^3,26–28^, suggesting a crucial role of bZIP transcription factors in root and root nodulation. Despite these research progress in bZIP TFs, neither the evolution of TGA TFs nor their roles in defence and symbiosis are characterized in leguminous plants. In this study, we identified 174 bZIP TFs in the soybean genome including 25 homologs of AtTGAs, GmTGAs. A sub-clade of the GmTGAs fall into a unique clade of leguminous TGAs which exhibit unique properties in their protein structures. *GmTGAs* are differentially expressed in different organs in response to rhizobial infection and nitrogen. Our findings shed light on the possible roles of *GmTGAs* in plant growth and symbiotic nodulation in soybean in response to nitrogen conditions.

### TGA TFs in soybean evolved differently from *Arabidopsis* TGAs

bZIP protein family is a large family of plant transcription factors and is highly conserved in plants with diverse functions^29,30^. Different plant species have maintained a different number of bZIP TFs during evolution. *Arabidopsis* has 75 members of this family, while the numbers of bZIP family exceed 100 in some species^31^. In soybean, the number of bZIP TFs remains controversial. An earlier study reported a total of 120 and 131 soybean bZIP members^32,33^ while recent reports found 138 or 160 bZIP genes in the soybean genome^24,34^. In this study, we identified a total of 174 bZIP TFs in soybean from the soyKB database (Fig. 1). The differences in the total number of soybean bZIP TFs is mainly due to the continuous updates in the databases and the different approaches used by the authors.

TGAs are members of the bZIP TF family and mediate a range of processes including plant defence and growth through regulating transcription of the downstream genes^35^. We identified a total of 25 *GmTGA*s in soybean with two as possible pseudogenes (Fig. 3). The soybean genome was reported to have an average of 2.55 duplication per segment, suggesting at least one original genome tetraploidization^36^. Two independent duplications in soybean approximately 59 and 13 million years ago resulted in the presence of 75% of the genes in multiple copies^37^. It is conceivable that soybean has 130% more *GmTGA*s than *Arabidopsis*. The fact that there are varied putative orthologs for each *AtTGA* (Fig. 3) supports the notion that nature has favoured some of the *GmTGAs* more than the others for better survival with the fluctuations in the environment during the evolutionary history of soybean. Likewise, the extra copies of the *GmTGA*s, in particular, the soybean specific *GmTGA*s, may have evolved to survive better and meet the high demand for nitrogen and symbiotic nitrogen fixation during the evolution.

It is well known that *AtTGA*9 and *AtTGA10* are involved in reproductive growth and plant defence in *Arabidopsis*^38,39^. In our study, the response of the selected putative orthologs of *AtTGA9* and *AtTGA*10 to rhizobial infection in soybean provides evidence of their novel function as symbiotic nodulation regulators (Figs 6 and 8). Also, the soybean orthologues of *AtTGA8* (also known as PAN) that has been recognized as an essential regulator of floral organ number and quiescent centre (QC) in *Arabidopsis*^40^ seems to have also gotten favour during evolution. There are three orthologues (*GmTGA1*, *GmTGA2 and GmTGA10*) of *PAN* found in soybean (Fig. 3), and in addition to the expression of *GmTGA1 and GmTGA2* in roots, they were also expressed highly in nodules and response to nitrate (Figs 7 and 8). The two *GmTGAs* (*GmTGA1 and GmTGA2*) that expressed highly in nodules are born more recently than *GmTGA10* (closely related to *AtPAN*) through gene duplication, have possibly gone through neofunctionalization (Fig. 3). A previous study has reported *GmTGA1* and *GmTGA2* as nodules specific genes in a transcriptomic study in soybean^25^. These findings strongly suggest that these *GmTGA* genes might have gained new functions regulating plant growth and responses to abiotic factors and microorganisms including rhizobial symbiosis through different molecular mechanisms.

### Involvement of *GmTGA*s in the integration of defence, nitrogen, and nodulation

Soybean is a high nitrogen demand crop. It has evolved highly complicated regulatory networks to sense nitrogen and to differentiate the friendly bacteria from pathogens under low nitrogen so that the symbiosis relationship can be established. Therefore, integration of nitrogen sensing, immune signalling, and nodulation signalling are essential for precise and dynamic regulation of nodulation. Although TGAs were originally found for their crucial roles in plant defence system^9,41^, recent studies have revealed that their roles are not limited to immune response^42,43^. The TGAs, as transcription factors, probably can target genes in various signalling pathways that modulate plant growth and responses to abiotic and biotic stimuli. In this study, we proposed multiple roles of the *GmTGAs* in nitrogen response and nodulation. Among the tested genes, many genes are responsive to rhizobial infection, some of them respond to nitrogen, and some are regulated by both nitrogen availability and rhizobial inoculation (Figs 6 and 7). Although the levels of expression or expression patterns of these *GmTGA* genes varied in response to the treatments or in different organs, it is highly likely that these genes are involved in either nitrogen or rhizobia or both in soybean.

During rhizobial infection at low nitrogen conditions, rhizobia are initially considered as pathogens, and the plants activate the immune system against rhizobial infection before recognition^44^. The data that *GmPR1* was first up-regulated by rhizobia inoculation and were then restored to the basal expression level in the root (Fig. 6) support the hypothesis. This is also mainly in agreement with the previous studies about the immune-related gene expression during rhizobial infection in soybean^45,46^. It has been proposed that down-regulation of *PR1* and suppression of immunity may be due to reactive oxygen species (ROS) that are produced during symbiosis^47^. Based on the data that the several orthologs of *AtTGAs* showed a similar trend to *GmPR1* expression in response to rhizobia, we hypothesized that these *GmTGA*s might be coordinately involved in transcription regulation of *GmPR1*. It is possible that rhizobia activate *GmTGA*s-*GmPR1* module at the initial stage which then is repressed by activation of nodulation signalling allowing rhizobial entrance. The rest of rhizobial responsive *GmTGA*s may participate in the immune response by other *GmTGA*s or other biological processes during rhizobial infection. It is also possible that the induction of some of the immune genes *(GmTGAs)* is co-opted for symbiosis purposes to avoid plant counter-attack, a theory proposed somewhere else^48^. Interestingly, we found that *GmPR1* was significantly downregulated and eleven *GmTGAs* were upregulated by high nitrate (Fig. 7), while three *GmTGAs* were slightly downregulated (Supplementary Fig. S6b). It is likely that high nitrate may suppress the immune system through these *GmTGA*s-mediated repressions of *GmPR1*. It has been reported that nitrogen availability influences plant disease^49^. It will be interesting to uncover the molecular mechanisms of how *GmTGA*s mediate interplay among nitrogen and rhizobia/immune response signalling pathways. Functional analysis of the *GmTGA*s that are expressed in different organs will also provide novel insights into the regulatory roles of these *GmTGA*s in plant growth and organ responses to nitrogen, rhizobial inoculation and symbiosis. Considering the crucial role of PR1 and TGA in SA-induced plant defence system and the function of SA in legume symbiotic nodulation, it will be of great significance to further elucidate the molecular mechanism of how the host’s defence system is changed during rhizobial infection and symbiotic nodulation in legumes.

### A group of TGAs is specific to legumes

The fact that GmTGAs are highly conserved with AtTGAs in typical domains and protein/gene structures (Supplementary Fig. S3) suggests that these GmTGAs may exert their function similarly to the AtTGAs. Interestingly, phylogenetic analysis of GmTGAs and AtTGAs reveal that the clade-VI was specifically comprised of three GmTGAs without AtTGAs, while the rest of six clades were comprised of both AtTGAs and GmTGAs (Fig. 3). This observation indicates that the legumes-specific TGAs have evolved during evolution. The further large scale of phylogenetic analysis using 39 members from 16 species from 12 genera has confirmed the specificity of the clade uniqueness to legumes (Fig. 4). All the three members in the soybean-specific clade were induced by rhizobial infection (Fig. 6) but the expression of *GmTGA4* in nodules at 21 DAI was much higher in comparison with *GmTGA3* and *GmTGA23*, suggesting their diverse roles in nodulation (Fig. 8; Supplementary Fig. S6). Among them, one (*GmTGA4*) and other two (*GmTGA3* and *GmTGA23*) were induced or suppressed by nitrate, respectively (Fig. 7; Supplementary Fig. S6). These results strongly suggest that the legumes-specific *TGAs* may have unique structures which are different from regular TGA proteins and may be involved in some legumes-specific biological processes, such as legumes-rhizobia symbiotic nodulation.

A detailed analysis of the sequences of three soybean-specific GmTGA proteins revealed that in addition to the common bZIP domains, two (GmTGA3 and GmTGA23) of three legume-specific TGA proteins contain one autophagy sequence in their proteins (http://bioinformatics.psb.ugent.be/beg/software)^22^. Further investigation found that the autophagy sequences were present in 32 out of 39 members of the legume-specific group, which strongly support the notion that these special TGAs are evolved specifically in legumes. Autophagy is a catabolic process that mediates the degradation of cell components or dangerous molecules in the cytoplasm^50^. It has been shown that autophagy plays both anti-microbial and pro-microbial roles in plant-microbe interaction^51^. In legumes, functional analysis of autophagy-related genes, such as PI3K and Bax inhibitor-1, have demonstrated that autophagy-related biological processes including defence, intracellular trafficking and autophagy are crucial for nodulation and nodule senescence^52^. The discovery of the unique property in GmTGA3 and GmTGA23 suggests that these GmTGAs might have gone through a gain-of-function event during evolution. Currently, we do not know what the biochemical and molecular functions of the autophagy sequences in these proteins are, and what the biological functions of these soybean or legumes-unique TGA proteins are. However, the presence of these sequences in the legumes-specific TGAs gives the proteins a unique identity among GmTGAs. Importantly, the presence of the autophagy sequence in these GmTGA proteins may implicate the biological roles of these proteins in soybean-rhizobia interaction and their potential biochemical or molecular functions that are associated with protein stability or integration with the autophagy process. Based on the gene expression pattern (Figs 6–8; Supplementary Fig. S6b), these soybean-specific *GmTGA* genes may participate in plant responses to nitrogen and rhizobia, but we do not exclude the possibility that these genes are involved in other biological processes in soybean.

In addition, one member (GmTGA4) of this clade is an orphan gene which is specific to soybean and has only one copy in the whole genome of soybean (https://bioinformatics.psb.ugent.be/plaza/versions/plaza_v3_dicots/workbench/subset/4304/sp/gma). Since soybean has some unique features or traits compared to other legumes, it is possible that *GmTGA4* may modulate these specific biological processes unique for soybean. The unveiling of the roles of these soybean-specific *GmTGA*s will provide new insights in the understanding of soybean specific biological processes including nodulation and will provide a molecular base for the importance of autophagy in soybean nodulation in specific and legume nodulation in general.

## Materials and Methods

### Sequence extraction and structure analysis

To find out the total number of bZIP TFs in the soybean genome, we used the browse function (browse gene family) of the soykb database and selected bZIP TF factors family (http://soykb.org/gene_family.php)^53^. A total of 366 variants of 175 transcription factors were found in the database. For the sake of simplicity, we selected only one variant of each gene for phylogenetic analysis, and amino acid (AA) sequences were downloaded from the soykb database. To find out the total number of TGA TFs among 175 bZIP family members in the soybean genome, AA sequences of *Arabidopsis* TGA TFs were downloaded from The *Arabidopsis* Information Resource (TAIR) (https://www.Arabidopsis.org). A total of 25 *GmTGA*s were identified based on grouping with *AtTGA*s and their deviation (multiple alignments) from other members of GmbZIP family members. All the 25 *GmTGAs* were named as *GmTGA1*, *GmTGA2* up to *GmTGA25* based on their position on chromosomes in the soybean genome. The information about the genes’ nomenclature is given in Supplementary Table S2. Distribution of a total number of genes and the total number of gene variants per chromosome in the soybean genome were subjected to excel for a graphical representation.

### Phylogenetic and bioinformatics analysis

All the bZIP sequences along with AtTGAs were subjected to Geneious software for phylogenetic analysis, and all those sequences that clad with AtTGAs were considered as GmTGAs. The phylogenetic tree was constructed using MAFFT alignment and FastTree plugins in geneious. Sequences for the trees that only contain soybean and *Arabidopsis* were downloaded from soyKB and TAIR, respectively. For the construction of larger trees, the blast option of Geneious was used to retrieve the related sequences from NCBI. Duplicates and poorly aligned sequences were removed before constructing a tree. Gene duplication data was retrieved from Plaza 4.0 (https://bioinformatics.psb.ugent.be/plaza/), and conserved motifs were identified through MEME online suit (http://meme-suite.org/tools/meme) with the default setting^54^.

### Tissue level expression pattern analysis

Tissue-specific expression data of the selected 25 *GmTGA*s were downloaded from soybean eFP browser (http://bar.utoronto.ca/efpsoybean). The data were subjected to the “expression-based heat maps” tools of heat mapper, an online tool for converting numerical data into heatmaps (http://www2.heatmapper.ca/). Heat maps were built using row as scale type, clustering method as average linkage and distance measurement method as Euclidean. Data for total genes expressed in roots and nodules in inoculated plants were obtained from soykb tools “Heatmap and Hierarchical Clustering” (http://soykb.org/heatmap2/index.php)^53^. Orange was used as a tool to correlate the expression data and to present the data in a heatmap form^55^.

### Plant materials and treatments

Soybean seeds were surface sterilized with 75% alcohol and were grown in sterilized pots containing sterilized vermiculate and were watered with a nutrient solution previously used by Wang *et al*.^56^ with a modification that either no nitrogen (N_2_-free nutrient solution) or high nitrogen level was used (1.174 g L^−1^ KNO_3_). Seedlings were divided into three different trays and were raised independently and were grown under the conditions described somewhere^57^. Seven days after sowing, the seedlings of one tray were watered with high N_2_ nutrient solution. Control samples from root and shoot were taken just before treatment of the seedlings and were considered as 0 hours after treatment (HAT). The samples were immediately put into liquid N_2_ and were stored at −80 °C for subsequent RNA extraction. Samples were collected at 0, 3, 6, 12, and 24 HAT. The rest of the seedlings were grown under high nitrogen condition for 21 days. Seedlings of the second tray were inoculated with rhizobia (OD = 0.08 and 30 mL plant^−1^) as proposed^58^, and samples were collected at five-time points for RNA extraction. Samples immediately taken before inoculation were considered as 0 hours after inoculation (HAI). Roots and shoots samples were collected at 0, 3, 6, 12, and 24 HAI, and were frozen immediately in liquid N_2_ and stored at −80 °C for subsequent RNA extraction. The remaining seedlings treated with rhizobia along with no treated seedling (3^rd^ tray) were grown until 21 days after inoculation under nitrogen-free (N-Free) nutrient solution. Roots, leaves, and nodules samples were collected 21 days after inoculation (DAI) and were stored at −80 °C for RNA extraction.

### RNA extraction and quantitative PCR analysis

The frozen samples were taken back from −80 °C and were crushed into fine powder. RNA set was extracted using Trizol reagent (Tiangen Biotech [Beijing] Co., Ltd., Beijing, China) and then treated with gDNA digester (Tianjin Novogene Bioinformatics Technology Co., Ltd. www.novogene.com) to remove the leftover genomic DNA. First-strand cDNA was synthesized from the total RNA using Honor^TM^ II 1^st^ Strand cDNA Synthesis SuperMix for qPCR (gDNA digester plus) kit provided by Novogene. qRT-PCR was performed using Unique Aptamer^TM^ qPCR SYBR^®^ Green Master Mix (No Rox) provided by Novogene (Tianjin Novogene Bioinformatics Technology Co., Ltd. www.novogene.com). Samples were collected from three independent biological replication and qRT-PCR was performed on two technical repeats from each biological replication. The Melt-curve analyses were performed using the conditions previously used^59,60^. *GmELF1B* was used as the internal reference gene^61^. Specific primers used for determining the expression level of the selected *GmTGAs* in time series samples of treated plants are listed in Table S3.

### Statistical analysis

The qRT-PCR numeric values for gene expression were subjected to GraphPad Prism (version 7.00 for Windows, GraphPad Software, La Jolla California USA, www.graphpad.com) for graphical representation. The error bars are based on the standard error of mean calculated from the replicated data. Pearson correlation was used for correlating the expression data among *GmTGA*s and different tissues while k values were used for clustering the genes or tissues using Orange as a statistical package^55^. Gene expression data were analyzed through Analysis of Variance (ANOVA) independently for roots and shoots. Least significant differences (LSD) was used to compare the data taken at different time points for statistical significance. Statistix 8.1 was used for comparison.

## Supplementary information

Supplementary information

## Data Availability

All the data used in the study are made available either in the main text or in the supplementary information.
