# Supplementary material for: Genome-wide identification and evolutionary analysis of TGA transcription factors in soybean
Source: Sci Rep. 2019 Aug 1;9:11186. doi: 10.1038/s41598-019-47316-z (PMC6672012; doi:10.1038/s41598-019-47316-z)
Supplement: Supplementary file 1 — Supplementary information [file 41598_2019_47316_MOESM1_ESM.docx]

**Genome-wide identification and evolutionary analysis of TGA transcription factors in soybean**

**Supplementary Information**

[Ihteram Ullah](javascript:enjoy();)^1,2,3^, Mahmoud Magdy^4,5^, Lixiang Wang^6^, Mengyu Liu^1^, Xia Li^3§^

^1^ Center for Agricultural Resources Research, Institute of Genetics and Developmental Biology, Chinese Academy of Sciences, Shijiazhuang, China.

^2^ University of Chinese Academy of Sciences, Beijing, China.

^3^ State Key Laboratory of Agriculture Microbiology, College of Plant Science and Technology, Huazhong Agricultural University, Wuhan, 430070, Hubei Province, China.

^4^ Key laboratory of horticulture, plant biology, Huazhong Agricultural University, Wuhan, China

^5^ Genetics Department, Faculty of Agriculture, Ain Shams University, Cairo, Egypt.

^6^ School of biological and chemical engineering, Panzhihua University, Panzhihua, China

^§^Corresponding author: xli@mail.hzau.edu.cn.


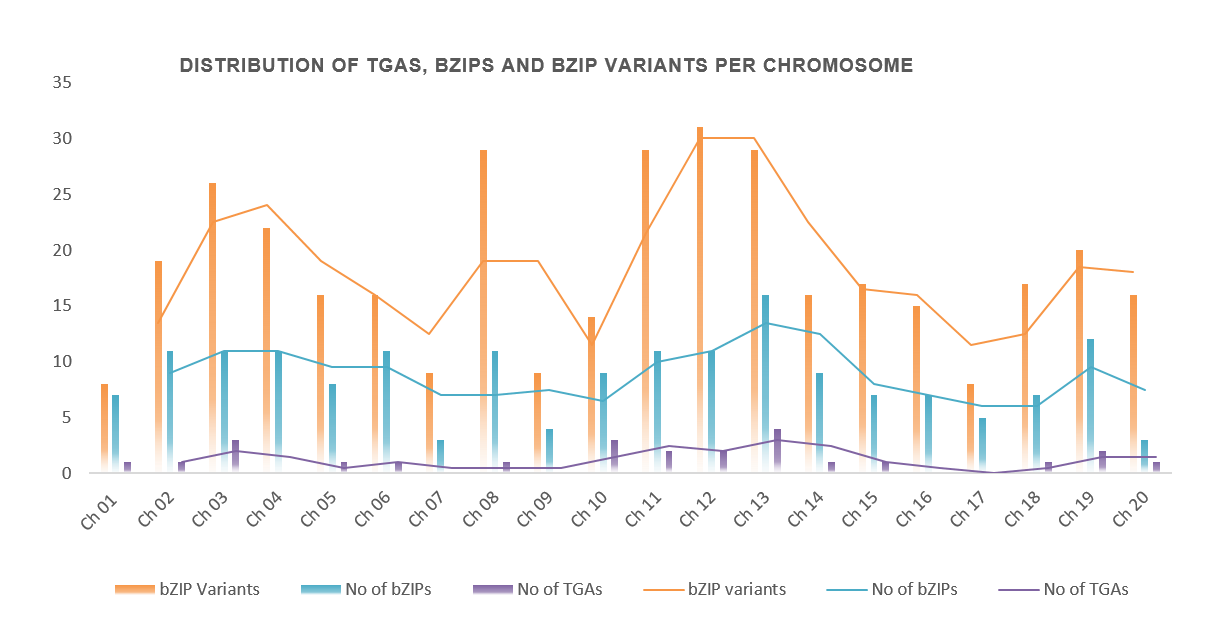


**Figure S1.** Distribution of bZIP variants, bZIP TFs, and GmTGAs across 20 chromosomes of soybean genome.


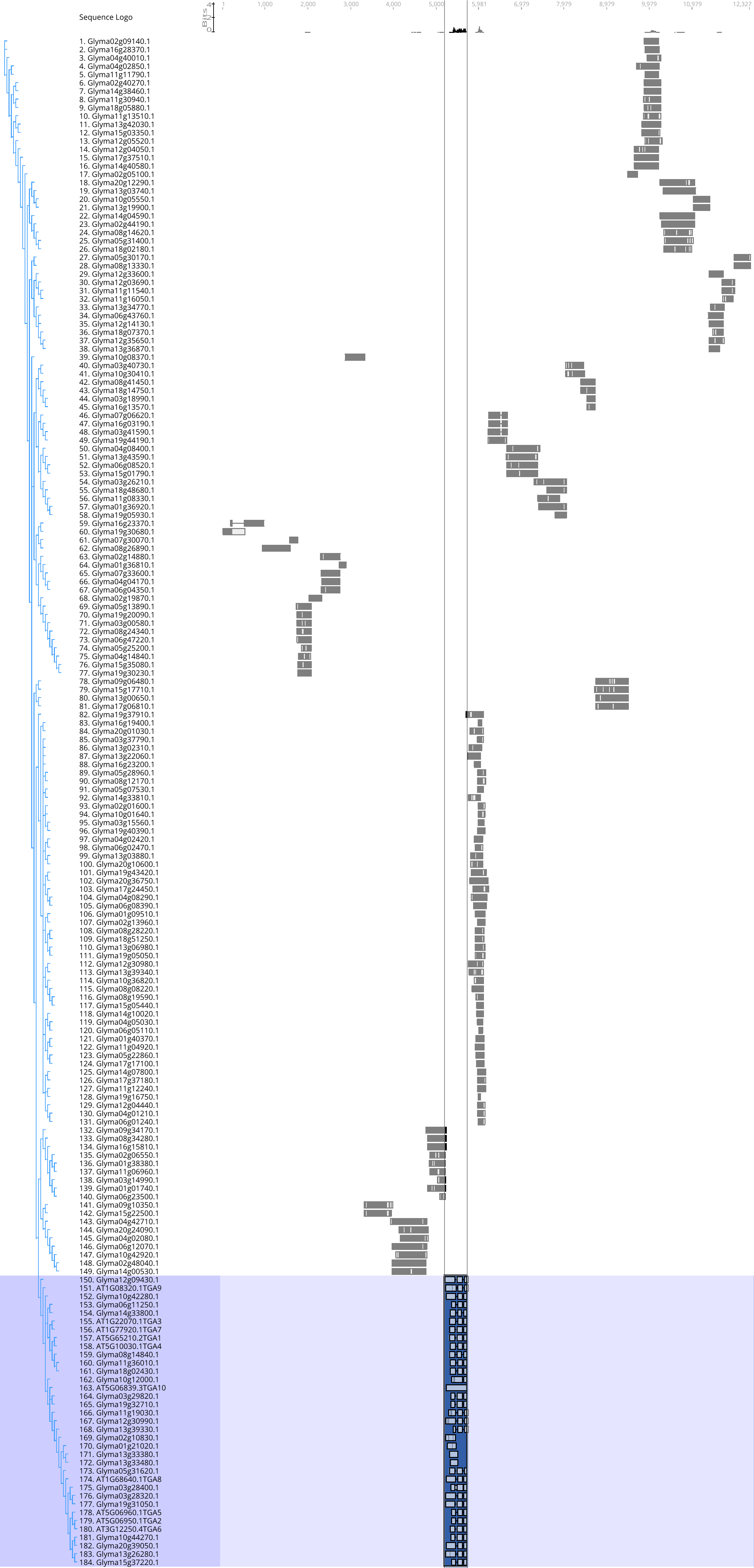


**Figure S2.** Alignment of the 174 GmTGAs with 10 AtTGAs. The shaded groups are the groups containing GmTGAs and AtTGAs. GmTGAs and AtTGAs are aligned separately from the other groups of bZIP TFs


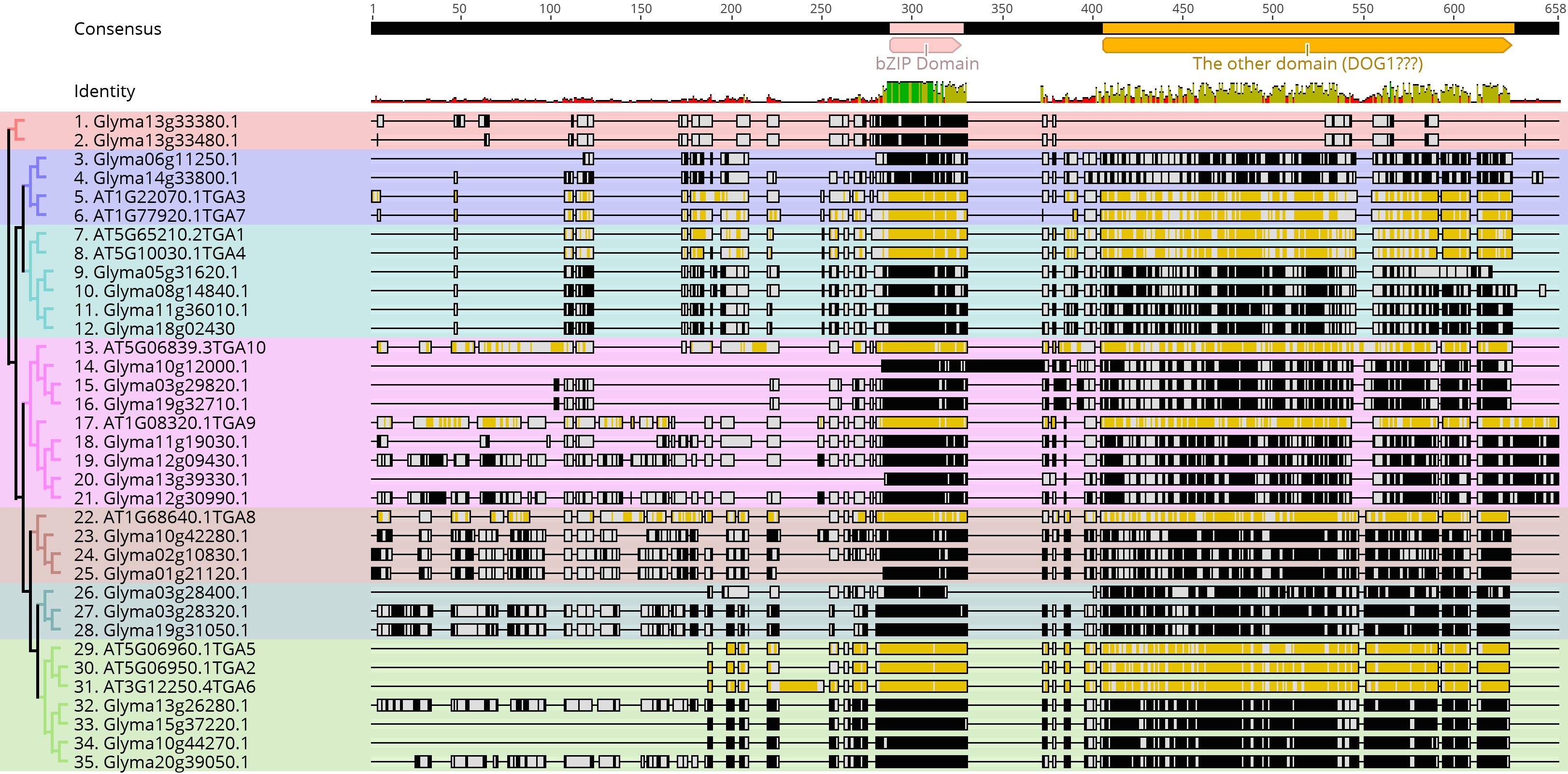


**Figure S3.** The phylogeny and sequence alignment of GmTGAs and AtTGAs. Twenty-five GmTGAs in black and white and 10 AtTGAs in yellow and white were aligned. bZIP domain and the other domains were shown in different colors along the consensus line at the top of the alignment. The dark regions showed the similarities, while the light regions showed the differences among the TGAs.


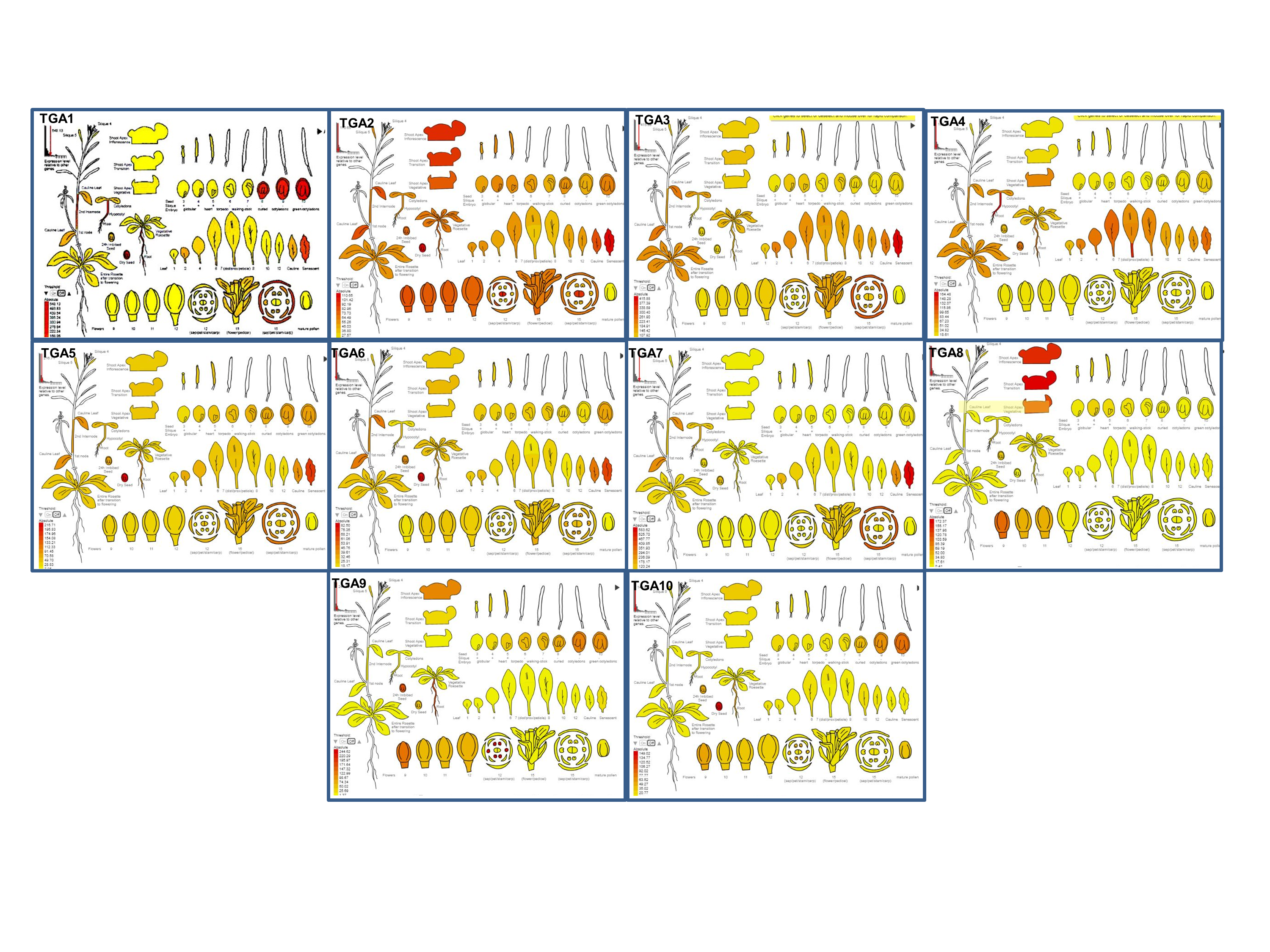


**Figure S4.** Expression patterns of *AtTGAs*. Majority of the *AtTGAs* were expressed highly in aerial parts. The data were retrieved from Arabidopsis eFP browser accessed Feb 20, 2019 (<http://bar.utoronto.ca/efp/cgi-bin/efpWeb.cgi>)^62^.


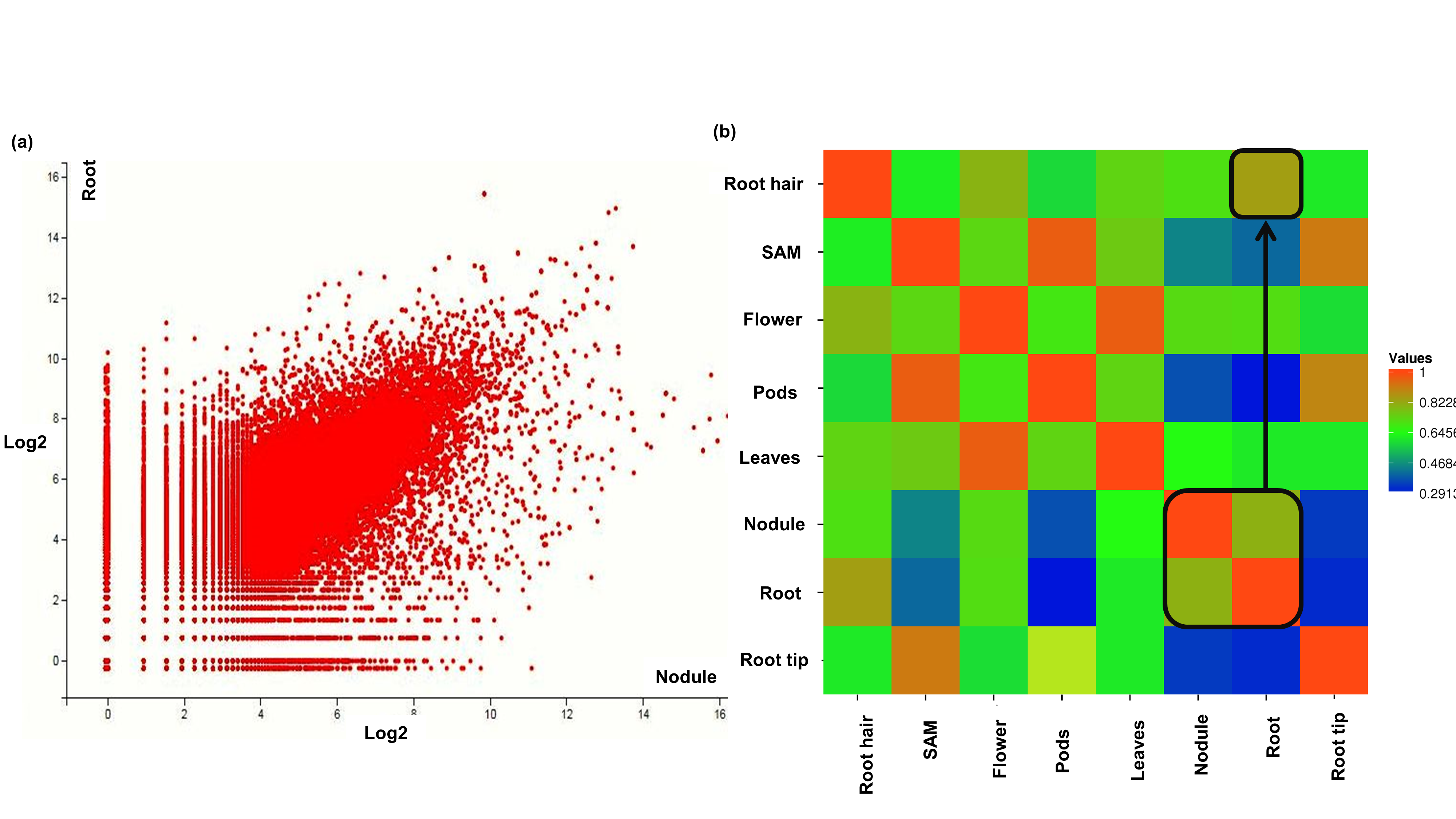


**Figure S5.** Co-expression of genes in roots and nodules. a) The total number of genes expressed in both in roots and nodules under symbiosis. Most of the genes were co-expressed in roots and nodules except few genes, which were expressed either in roots or in nodules. The scatter plot is retrieved from soyKG^53,63,64^. b) Correlation among the 25 *GmTGAs* based on their expression in different tissues. The expression was highly correlated in roots, nodules and root hairs.


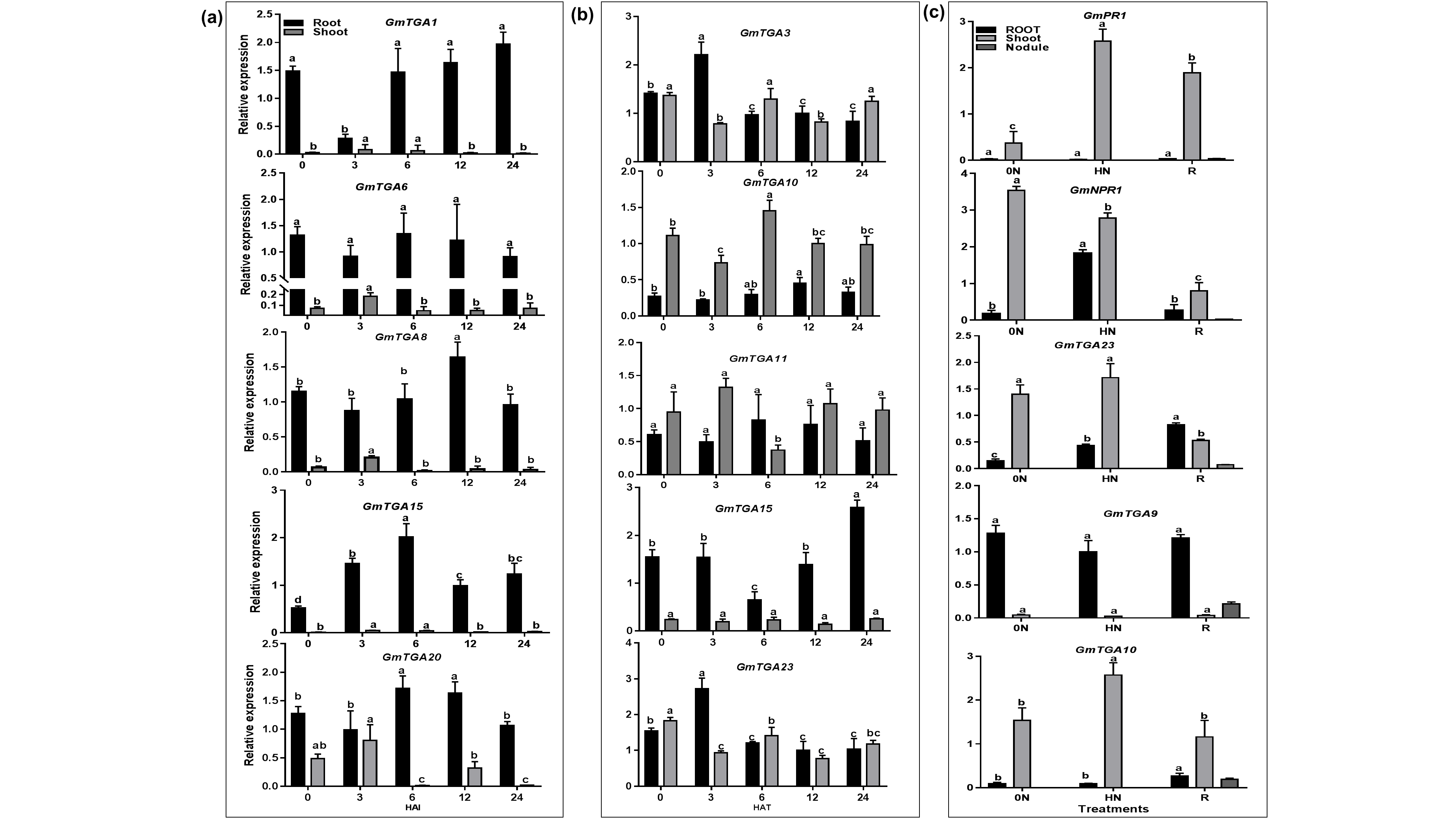


**Figure S6.** Response of *GmTGAs* to rhizobia and nitrate treatments. Soybean plants were grown under no nitrate and high nitrate without rhizobia inoculation and no nitrate with rhizobia inoculation, and the gene expression of *GmTGAs* in root was analyzed at the specified time points after rhizobia inoculation (a) and high nitrate (b); (c) Expression *GmPR-1*, *GmNPR-1* and *GmTGAs* in nodules at 21 DAI in soybean. HAI is hours after inoculation; HAT stands for hours after treatment. 0N=zero nitrogen treatment, HN=high nitrogen treatment, R=rhizobia treatment

**Table S1.** List of the duplicated *GmTGA* genes in soybean

| **Gene ID** | **Type** | **Gene name** | **Duplicates** |
| --- | --- | --- | --- |
| GM03G28320 | coding | Glyma03g28320 | GM19G31050 |
| GM03G28400 | coding | Glyma03g28400 | GM03G28320 |
| GM06G11250 | coding | Glyma06g11250 | GM13G02351, GM14G33800, GM04G43411, GM0G14840, GM05G31623 (Glyma05G31620) |
| GM08G14840 | coding | Glyma08g14840 | GM05G31623 (Glyma05G31620), GM18G02430, GM06G11250, GM11G36010 |
| GM10G42280 | coding | Glyma10g42280 | GM20G24766, GM01G21120 |
| GM10G44270 | coding | Glyma10g44270 | GM20G39050 |
| GM11G36010 | coding | Glyma11g36010 | GM18G02430, GM08G14840, GM05G31623 |
| GM12G09430 | coding | Glyma12g09430 | GM13G39325, GM11G19026, GM12G30990 |
| GM12G30990 | coding | Glyma12g30990 | GM13G39325 (Glyma13G38330), GM12G09430, GM11G19026 (Glyma11G319030) |
| GM13G26280 | coding | Glyma13g26280 | GM15G37220 |
| GM13G33380 | coding | Glyma13g33380 | GM13G33380 |
| GM13G33480 | coding | Glyma13g33480 | GM13G33380 |
| GM14G33800 | coding | Glyma14g33800 | GM04G43411, GM13G02351, GM06G11250, GM18G02430 |
| GM15G37220 | coding | Glyma15g37220 | GM13G26280 |
| GM18G02430 | coding | Glyma18g02430 | GM08G14840, GM13G02351, GM11G36010, GM05G31623 (Glyma05G31620), GM14G33800 |
| GM19G31050 | coding | Glyma19g31050 | GM03G28320 |
| GM20G39050 | coding | Glyma20g39050 | GM10G44270 |

**Table S2.** Nomenclature of the *GmTGAs*, their IDs and relationship to *AtTGA* clades

| **Gene names** | **SoyKB Gene IDs** | **Phytozome Gene IDs** | **Phytozome Gene Alias** | **Corresponding AtTGA clades** |
| --- | --- | --- | --- | --- |
| ***GmTGA6*** | *Glyma05g31620* | *Glyma.05G182500* | *Glyma05g31623* | **AtTGA clade-I** |
| ***GmTGA8*** | *Glyma08g14840* | [*Glyma.08g140100*](https://www.soybase.org/sbt/search/search_results.php?category=FeatureName&version=Glyma2.0&search_term=Glyma.08g140100) | *Glyma08g14840* | **AtTGA clade-I** |
| ***GmTGA13*** | *Glyma11g36010* | [*Glyma.11g236300*](https://www.soybase.org/sbt/search/search_results.php?category=FeatureName&version=Glyma2.0&search_term=Glyma.11g236300) | *Glyma11g36010* | **AtTGA clade-I** |
| ***GmTGA22*** | *Glyma18g02430* | [*Glyma.18g020900*](https://www.soybase.org/sbt/search/search_results.php?category=FeatureName&version=Glyma2.0&search_term=Glyma.18g020900) | *Glyma18g02430* | **AtTGA clade-I** |
| ***GmTGA11*** | *Glyma10g44270* | [*Glyma.10g296200*](https://www.soybase.org/sbt/search/search_results.php?category=FeatureName&version=Glyma2.0&search_term=Glyma.10g296200) | *Glyma10g44270* | **AtTGA clade-II** |
| ***GmTGA16*** | *Glyma13g26280* | [*Glyma.13g193700*](https://www.soybase.org/sbt/search/search_results.php?category=FeatureName&version=Glyma2.0&search_term=Glyma.13g193700) | *Glyma13g26280* | **AtTGA clade-II** |
| ***GmTGA21*** | *Glyma15g37220* | [*Glyma.15g232000*](https://www.soybase.org/sbt/search/search_results.php?category=FeatureName&version=Glyma2.0&search_term=Glyma.15g232000) | *Glyma15g37220* | **AtTGA clade-II** |
| ***GmTGA25*** | *Glyma20g39050* | [*Glyma.20g246400*](https://www.soybase.org/sbt/search/search_results.php?category=FeatureName&version=Glyma2.0&search_term=Glyma.20g246400) | *Glyma20g39050* | **AtTGA clade-II** |
| ***GmTGA7*** | *Glyma06g11250* | [*Glyma.06g107300*](https://www.soybase.org/sbt/search/search_results.php?category=FeatureName&version=Glyma2.0&search_term=Glyma.06g107300) | *Glyma06g11250* | **AtTGA clade-III** |
| ***GmTGA20*** | *Glyma14g33800* | [*Glyma.14g167000*](https://www.soybase.org/sbt/search/search_results.php?category=FeatureName&version=Glyma2.0&search_term=Glyma.14g167000) | *Glyma14g33800* | **AtTGA clade-III** |
| ***GmTGA5*** | *Glyma03g29820* | *Glyma.03G142400* | *Glyma03g29825* | **AtTGA clade-IV** |
| ***GmTGA9*** | *Glyma10g12000* | *Glyma.10G092100* | *Glyma10g11985* | **AtTGA clade-IV** |
| ***GmTGA12*** | *Glyma11g19030* | *Glyma.11G183700* | *Glyma11g19026* | **AtTGA clade-IV** |
| ***GmTGA14*** | *Glyma12g09430* | [*Glyma.12g088700*](https://www.soybase.org/sbt/search/search_results.php?category=FeatureName&version=Glyma2.0&search_term=Glyma.12g088700) | *Glyma12g09430* | **AtTGA clade-IV** |
| ***GmTGA15*** | *Glyma12g30990* | [*Glyma.12g184500*](https://www.soybase.org/sbt/search/search_results.php?category=FeatureName&version=Glyma2.0&search_term=Glyma.12g184500) | *Glyma12g30990* | **AtTGA clade-IV** |
| ***GmTGA19*** | *Glyma13g39330* | *Glyma.13G316900* | *Glyma13g39325* | **AtTGA clade-IV** |
| ***GmTGA24*** | *Glyma19g32710* | *Glyma.19G145300* | *Glyma19g32715* | **AtTGA clade-IV** |
| ***GmTGA1*** | *Glyma01g21020* | *Glyma.01G084200* | *Glyma01g21120* | **AtTGA clade-V** |
| ***GmTGA2*** | *Glyma02g10830* | *Glyma.02G097900* | *Glyma02g10833* | **AtTGA clade-V** |
| ***GmTGA10*** | *Glyma10g42280* | [*Glyma.10g276100*](https://www.soybase.org/sbt/search/search_results.php?category=FeatureName&version=Glyma2.0&search_term=Glyma.10g276100) | *Glyma10g42280* | **AtTGA clade-V** |
| ***GmTGA3*** | *Glyma03g28320* | [*Glyma.03g127600*](https://www.soybase.org/sbt/search/search_results.php?category=FeatureName&version=Glyma2.0&search_term=Glyma.03g127600) | *Glyma03g28320* | **Legume specific** |
| ***GmTGA4*** | *Glyma03g28400* | [*Glyma.03g128200*](https://www.soybase.org/sbt/search/search_results.php?category=FeatureName&version=Glyma2.0&search_term=Glyma.03g128200) | *Glyma03g28400* | **Legume specific** |
| ***GmTGA23*** | *Glyma19g31050* | [*Glyma.19g130200*](https://www.soybase.org/sbt/search/search_results.php?category=FeatureName&version=Glyma2.0&search_term=Glyma.19g130200) | *Glyma19g31050* | **Legume specific** |
| ***GmTGA17*** | *Glyma13g33380* | Not Available | Not Available | **Pseudo-gene** |
| ***GmTGA18*** | *Glyma13g33480* | Not Available | Not Available | **Pseudo-gene** |

**Table S3.** The primers used in this study

| Gene ID | Primer | (5' to 3') |
| --- | --- | --- |
| Glyma15g062400  (GmPR1) | F | TGCAAGATCAGAGGTGGGTGT |
|  | R | TATTGGCCACCACCACCAGA |
| Glyma09g064700 (GmNPR1) | F | GTGGAAGAGTGAAGCCTTTGCC |
|  | R | AGTAGATGTCCAGCATTGCTGC |
| Glyma11g36010 | F | TGCTGCTGCAAAAGCAGATGTT |
|  | R | TCAAACGCTGTTGCTCCGTC |
| Glyma18g02430 | F | CAAGGTATGGACAAACTCCGGC |
|  | R | CTGCCGAAGATGATCAGCCTG |
| Glyma03g28400 | F | GAAACTGATATCGCCAGCAGCC |
|  | R | TGCTGCAATAGCTTTCAGCCTG |
| Glyma19g32710 | F | ATGAGGCCTCCAACTCTCAACA |
|  | R | GACCTTTGCGGTTGCTCTCTC |
| Glyma14g33800 | F | AAGTGAACATGTGGGGGGACA |
|  | R | ATTTCCGAGCAGCCTCACGAT |
| Glyma10g42280 | F | TATCACCGGGGAAATGACCCC |
|  | R | ACAACAGCCACATTGCTCGAC |
| Glyma02g10830 | F | AGGCTACACACTTTCCCTCCT |
|  | R | GAGTCGCTGCAGAATGAACACT |
| Glyma01g21120 | F | CTGCTGAAGCCTCAACCTCTTC |
|  | R | TTGGTGTGGCTTCCACATCTG |
| Glyma05g31620 | F | TGGAAACTGGGTGGAGGAACA |
|  | R | GCTGCAGCAGATTTCATGCCA |
| Glyma08g14840 | F | TCTTGCACAAAATCGAGAGGCC |
|  | R | TAGCTGCACGAGCTTCAAACG |
| Glyma10g12000 | F | GAGCATGGTGGCCAAAATCGA |
|  | R | TTGTTGCTCCGTAAGAGGCTCA |
| Glyma12g30990 | F | ATGGCGAGCCACAGAATAGGA |
|  | R | CAATTGCTTCCTCCAGCTCCC |
| Glyma03g28320 | F | GGACACTGACAAGGTTCTGAACG |
|  | R | TATCAGCACCTAGAGCTTGGCT |
| Glyma19g31050 | F | TCTGAACGGGATGCCAAGCTA |
|  | R | ATCAGCACCTAGAGCTTGGCT |
| Glyma10g44270 | F | GCTGACGCCAGTCCTAGAACT |
|  | R | AAGCCTCCGGAGACTCTTCTG |

**Reference list**

53. Joshi, T., Wang, J., Zhang, H., Chen, S. & Zeng, S. The evolution of soybean knowledge base (soyKB) trupti. *Plant Genomics Databases* **1533**, 149–159 (2017).

63. Winter, D. *et al.* An “Electronic Fluorescent Pictograph” browser for exploring and analyzing large-scale biological data sets. *PLOS ONE* **2** (8): e718 (2007).

63. Joshi, T. et al. Soybean knowledge base (SoyKB): a web resource for integration of soybean translational genomics and molecular breeding. *Nucl. Acids Res.* **42** (D1): D1245-D1252 (2014)

64. [Joshi, T. *et al.* Soybean Knowledge Base (SoyKB): a web resource for soybean translational genomics. *BMC Genomics.* **13** Suppl 1: S15 (2012)](http://www.biomedcentral.com/1471-2164/13/S1/S15)
